# Age does not affect sex effect of conditioned pain modulation of pressure and thermal pain across 2 conditioning stimuli

**DOI:** 10.1097/PR9.0000000000000796

**Published:** 2019-12-24

**Authors:** Joseph L. Riley, Yenisel Cruz-Almeida, Roland Staud, Roger B. Fillingim

**Affiliations:** a Department of Community Dentistry, College of Dentistry, University of Florida, Gainesville, FL, USA; b Pain Research and Intervention Center of Excellence, Clinical and Translational Science, Institute, Gainesville, FL, USA; c Department of Aging and Geriatric Research, University of Florida, Gainesville, FL, USA; d Department of Medicine, University of Florida, Gainesville, FL, USA

**Keywords:** Sex differences, Aging, Conditioned pain modulation, Conditioning stimulus, Test stimulus, CPM duration

## Abstract

**Introduction:**

Conditioned pain modulation (CPM) is a laboratory test resulting in pain inhibition through activation of descending inhibitory mechanisms. Older adults consistently demonstrate reduced CPM compared with younger samples; however, studies of sex differences in younger cohorts have shown mixed results.

**Objectives:**

This study tested for sex differences in CPM within samples of younger and older adults.

**Methods:**

Participants were 67 younger adults (mean age = 25.4 years) and 50 older adults (66.4 years). Study conditioning paradigms were the cold-pressor test and contact heat pain administered in separate sessions. Pressure pain threshold and ramping suprathreshold heat were the test stimuli across three time points after presentation of the conditioning stimuli (CS).

**Results:**

Significant inhibition was observed during both testing sessions. The hypothesis for sex differences across both age cohorts was supported only for ∆PPTh. However, sex differences did not reach significance for either paradigm using ascending suprathreshold heat as the test stimuli. The overall trend was that younger males experienced the strongest CPM and older females the weakest. From a methodological perspective, duration differences were seen in CPM, with inhibition decaying more quickly for PPTh than for suprathreshold heat pain. Furthermore, there were no differences in inhibition induced by cold-pressor test and contact heat pain as CS.

**Conclusion:**

Sex differences were similar across both age cohorts with males experiencing greater inhibition than females. Cross-sectional associations were also demonstrated between CPM inhibition and measures of recent pain, further supporting CPM as an experimental model with clinical utility.

## 1. Introduction

The experience of pain is a dynamic balance of excitatory and inhibitory endogenous modulatory mechanisms. Studies of pain processing systems frequently use laboratory protocols that engage pain inhibition.^25^ The phenomenon of diffuse noxious inhibitory controls is established in animal models and implicates the existence of an endogenous pain modulation system.^19,20^ The basic principle is “pain-inhibition-by-pain” where pain in a local area is inhibited by a second pain administered heterotopically.^40^ The term “conditioned pain modulation” (CPM) is used to refer to this phenomenon in humans and the resulting pain inhibition is thought to be a consequence of the activation of descending inhibitory mechanisms.^41^

A number of studies have shown that deficits in CPM are associated with a range of pain disorders, suggesting that a shift in balance between pain facilitation and pain inhibition is either antecedent to or the result of prolonged pain.^22,43^ Six studies have reported reduced pain inhibition associated with older age using protocols consistent with CPM.^6,10,18,31,32,39^ A larger number of studies have examined sex differences in CPM, typically in samples of healthy younger adults, but with mixed findings. A recent review concluded that about 40% of the publication have found males showing greater CPM than females.^15^ Few studies have compared the magnitude of sex differences across age cohorts.

This study tested the hypothesis that sex differences will be observed within samples of both younger and older adults using 2 well-validated laboratory pain modalities, the cold-pressor test (CPT) and contact heat pain (CHP) as conditioning stimuli (CS), in separate sessions. After consensus recommendations,^42^ 2 test stimuli (TS) have been used: an ascending thermal stimulus to pain at 40 (0–100) and pressure pain threshold (PPTh). An innovative aspect of the current methodology allowed testing the duration of CPM through 30 minutes across 4 combinations of stimuli and comparisons across 4 age–sex subgroups. We hypothesize that CPM would be significant at 3 and 15 minutes after presentation of the CS but not at 30 minutes. We also examined differences between the CPT and CHP to the foot, providing sensory input at the same spinal level and ascending–descending long tract activity. It was also hypothesized that significant associations between CPM and measures of recent pain will occur while adjusting for age and sex as a test of clinical relevance.

## 2. Materials and methods

### 2.1. Participants

Participants were 67 younger adults (age: mean = 25.4, SD = 6.8, range: 19–49 years; 36 females and 31 males) and 50 older adults (age: mean = 66.4, SD = 5.9, range: 56–77 years; 26 females and 24 males) and were balanced on race and ethnicity.

Interested individuals reviewed and signed an informed consent form. Eligibility was determined after completion of a health history questionnaire and interview. Study exclusion criteria included a Mini Mental Status score below 23,^8^ current use of narcotics or chronic use of analgesics, uncontrolled hypertension, systemic disease that restricts normal daily activities, neurological problems with significant changes in somatosensory perception, or serious psychiatric conditions. Recruits who received a grade 4 on the Graded Chronic Pain Scale (GCPS) (high disability-severely limiting) were excluded. The sampling strategy was to increase external validity by accepting that older adults may experience greater health issues as part of the aging process, but we eliminated individuals with disabling chronic pain for which loss of CPM is well established. The University of Florida Institutional Review Board approved the recruitment and study procedures.

### 2.2. Orientation and training session

Participants watched a video that described the study, the testing protocols, and rating pain in the 0 to 100 pain scale. Multiple training trials were administered using the study stimuli and sites while participants practiced using the pain rating system.

### 2.3. Testing sessions

This report used data collected during 2 sessions where only CPM testing was performed that occurred within the same calendar week with at least one washout day. No participants dropped out between CPM sessions; however, 5 participants discontinued after the orientation session. To start each session, participants relaxed in a comfortable chair for several minute, and were shown the video presentation seen during the orientation session.

**Figure 1.**
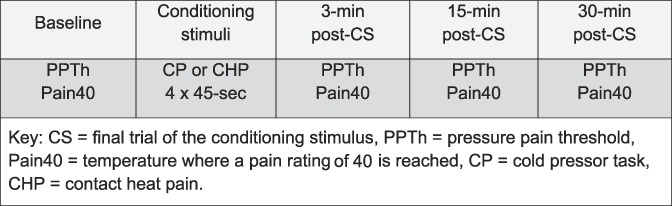
Timeline showing session progression.

Conditioned pain modulation was assessed using a within-session design with the order of the 2 CS sessions counterbalanced across participants. Two trials of PPTh followed by one trial of ascending contact heat stimuli to a pain rating of 40 (0–100 scale) were the study TS and were administered at baseline followed by administration of the conditioning stimulus (CS) assigned to that session. The CS consisted of the CPT or CHP, which were administered in 4 × 45-second trials with approximately 60 seconds between trials. Forty-five-second trials were used to ensure a robust inhibitory response among responders, reduce the potential for participants to withdraw early, and avoid vasovagal syncope. Participants were told that they may withdraw at any time by lifting their foot; however, none discontinued any trial. Subjects made a verbal rating of pain intensity on a 0 to 100 scale at 40 seconds during each CS trial that was averaged. The Situational Pain Catastrophizing Scale was administered verbally during the later breaks between CS trials. Post-CS trials of the TS occurred at 3, 15, and 30 minutes after the termination of the last CS trial (Fig. 1).

#### 2.3.1. Pressure pain threshold

A handheld Medoc Digital Pressure Algometer with a 10-mm diameter tip was used for the mechanical procedures. To assess PPTh, increasing pressure was applied centrally over the extensor/superior muscle group on the nondominant dorsal forearm at a rate of 0.25 kg per second. The participant was instructed to press a button when he or she first felt pain, at which time the device recorded the pressure in kilograms.

#### 2.3.2. Ascending suprathreshold heat pain (Pain40)

The thermal stimulus was delivered to the midpoint of the nondominant ventral forearm with a computer-controlled Medoc Pathway Thermal Sensory Analyzer (Ramat Yishai, Israel) using a 16 × 16-mm thermode. Pain was measured with an electronic visual analogue scale and a low-friction sliding potentiometer of 100-mm travel with the position of the slider electronically converted into a pain rating between 0 and 100. For the Pain40 measure, heat stimulus was applied in an ascending intensity (0.25°C per second) beginning at 43°C until a pain rating of 40 is achieved. The dependent variable was the temperature recorded at Pain40.

#### 2.3.3. Cold-pressor test

Participants immersed their dominant foot to the ankle into a water bath cooled by a Neslab refrigerated water circulator (Neslab, Portsmouth, NH). Water was continuously recirculated to prevent local warming and was maintained at a constant temperature. The water level was set at a height of 7 cm to keep the simulated area consistent. The temperature used for the first trial (8°C for males and 10°C for females) was adjusted on subsequent trials as needed to target pain ratings between 40 and 60.

#### 2.3.4. Contact heat pain

Thermal stimuli were delivered using the Medoc Pathway and the 16 × 16-mm thermode. The thermode was placed and held with moderate pressure on the bottom of the dominant foot immediately above the heel within the L4, L5 dermatome. The temperature used for the first trial (47°C for males and 46°C for females) was adjusted on subsequent trials as needed to target pain ratings between 40 and 60.

### 2.4. Self-report measures

The Situational Pain Catastrophizing Scale (S-PCS) is a 6-item adaptation of the Pain Catastrophizing Scale that assesses situation specific catastrophizing.^2^ This instrument asks about negative thoughts associated with the experimental pain just experienced. The Short-Form Health Survey-36 is a health survey that measures physical and psychological health.^37,38^ We adapted the time frame to the past 3 months. This study used the SF-36 Bodily Pain Scale (SF-36-BP) in which a higher score indicates less bodily pain. The GCPS is an 8-item scale that asks about pain severity and disability that results from pain and was designed for use in general population surveys and primary practice settings.^36^ Administration of the survey asked about pain in the past 3 months. The pain variables created for this study were the pain intensity and pain disability scores, pain duration, and the number of pain sites reported. In addition, the GCPS allows for the calculation of a chronic pain grade with 5 hierarchical categories: grades 0 (no pain) to 4 (high disability-severely limiting).

### 2.5. Statistical methods

SPSS version 23 was used for statistical analysis. Descriptive statistics were calculated for the study variables. Paired-samples *t*-tests were used to determine whether significant CPM occurred for each age–sex subgroup at each time point by comparing the baseline with the post-CS values. The study primary dependent variables, ∆PPTh and ∆Pain40, were calculated as the difference between the baseline and the post-CS values at each time point so that greater CPM is represented by a negative number.^41^ Interclass correlations or Pearson correlations were calculated within and across sessions for baseline PPTh and Pain40 and within and across sessions for ∆PPTh and ∆Pain40.

Age and sex differences for each of the outcome variables (∆PPTh and ∆Pain40) were tested using 4-way repeated-measures analyses of variance (ANOVAs). S-PCS scores were entered as covariates. PARADIGM (CPT, CHP) and TIME (3,15,and 30 minutes) were within-subject factors and AGE and SEX were between-subjects factors. For ease of presentation, nonsignificant effects are not described with the exception of the AGE × SEX interaction, which tested one of the study hypotheses. The Greenhouse–Geisser degrees of freedom adjustment was used when the assumption of sphericity was violated.^11^ A critical value of *P* = 0.05 was used as the cut point for interpretation of omnibus ANOVA effects. Next, correlations and multiple linear regression were used to test for associations between overall CPM and recent bodily pain. Conditioned pain modulation indices were created by averaging CPM across the 3 time points and creating CPT-∆Pain40 index, CHP-∆Pain40 index, CPT-∆PPTh index, and CHP-∆PPTh index. In step 1, age, sex, and the S-PCS were entered to adjust for differences. In step 2, the bodily pain score of the SF-36, the GCPS pain intensity and pain disability scores, pain days, and the number of pain sites were tested using the F to change statistic increase in *R*^2^.

## 3. Results

Mean and SD for the self-reported pain measures by age and sex are presented in Table 1. There were no differences across age group (*P* = 0.452) or sex (*P* = 0.631) in assignment to GCPS classifications^31^ (overall frequencies: 0-no pain = 40%, 1-low intensity = 36%, 2-high intensity = 15%, 3-moderately limiting = 9%). Mean and SD for the temperature and pain ratings for the CPT and CHP by age and sex are presented in Table 2. For the CPT, age (*P* = 0.09) and sex differences (*P* = 0.06) in the temperature approached significance. For the CHP, the mean temperature for the younger adults was lower than that for the older group (*P* = 0.044). No age or sex differences were seen for the pain intensity during the CS for either CPM session.

**Table 1:**
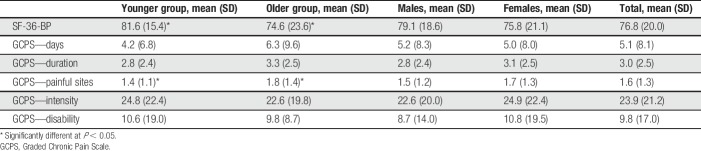
Mean and SD for the self-reported pain measures by age and sex.

**Table 2:**
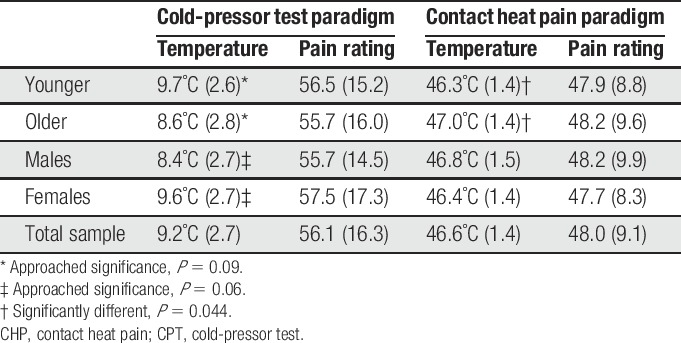
Mean and SD for the temperature and pain ratings by age and sex for both the CPT and CHP models.

Interclass correlations between baseline values for both PPTh (*r* = 0.80) and Pain40 (*r* = 0.72) were significant at *P* < 0.001, suggesting good reliability for the study TS.^4^ Correlations between baseline TS within-session (PPTh − Pain40) were *r* = 0.40 and *r* = 0.43 for CPT and CHP, respectively.

Mean and SD for ∆PPTh and ∆Pain40 at each time point are presented in Tables 3 and 4 by age and sex. The results of paired-samples *t*-tests between baseline and post-CS values are also presented in Tables 3 and 4 for each subgroup at each time point. ∆PPTh and ∆Pain40 were not associated with the CS testing temperatures or the pain experienced during administration of the CPT or CHP (*P* > 0.05). However, the S-PCS was significantly correlated with ∆PPTh during the CPT and ∆Pain40 for both the CPT and CHP and was used as a covariate in testing the study hypotheses. Correlations between the CPM experienced during CPT and CHP trials for ∆PPTh and ∆Pain40 are presented in Table 5.

**Table 3:**
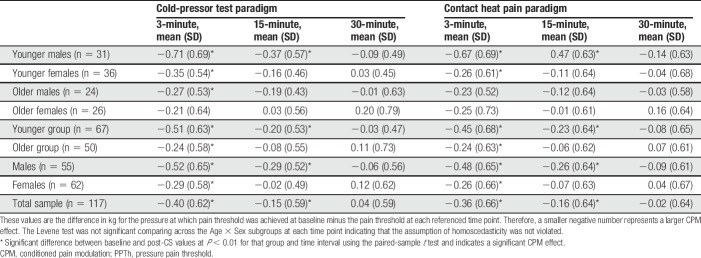
Unadjusted mean and SD for ∆PPTh for each time point by age and sex groupings.

**Table 4:**
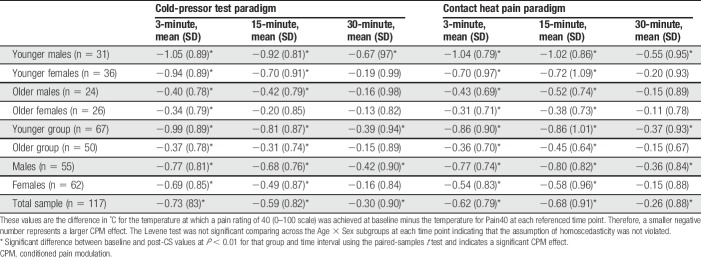
Unadjusted mean and SD for ∆Pain40 for each time point by age and sex groupings.

**Table 5:**
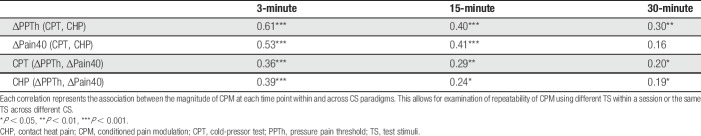
Correlations within and across sessions at each time point for test stimuli.

### 3.1. Age and sex differences in conditioned pain modulation using analysis of variance

**Figure 2.**
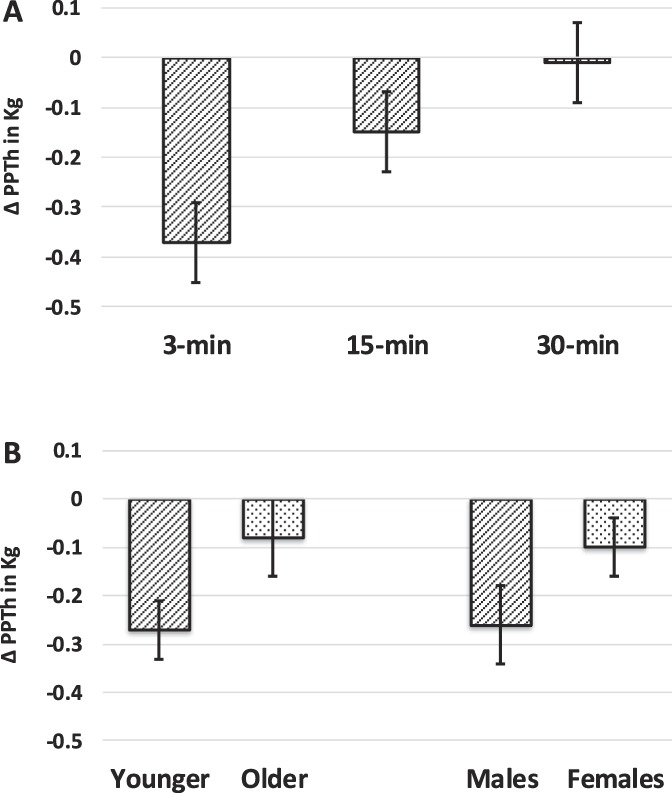
Adjusted mean and SE for ∆PPTh across time (A) and by age and by sex (B) across time collapsed across CPM paradigms. The TIME (*P* < 0.001), AGE (*P* = 0.041), and SEX (*P* = 0.044) main effects were significant. Conditioned pain modulation decreased significantly from the 3-minute (mean = −0.37) to 15-minute (−0.15) time points and was not significant at the 30-minute time point (0.11). Overall, younger adults experiencing significantly greater inhibition (−0.26) compared to the older group (0.06) and males (−0.26) exhibited significantly greater CPM than females (−0.08). CPM, conditioned pain modulation; PPTh, pressure pain threshold.

For ∆PPTh, the TIME [F(1.643,111) = 17.625, *P* < 0.001], AGE [F(1,111) = 5.623, *P* = 0.041], and SEX [F(1,111) = 5.459, *P* = 0.044] effects were significant. The AGE × SEX interaction was not significant. Conditioned pain modulation decreased from 3 minutes (mean = −0.37, confidence interval [CI] = −0.48 to −0.26) to 15 minutes (−0.15, 95% CI = −0.24 to −0.05), and was not significant at 30 minutes (0.01, 95% CI = −0.80 to 1.07). When collapsed across time, younger adults experienced greater inhibition (−0.26, 95% CI = −0.37 to −0.15) when compared with the older group (0.08, 95% CI = −0.21 to 0.09). Furthermore, males (−0.26, 95% CI = −0.39 to −0.13) exhibited greater CPM than females (−0.10, 95% CI = −0.20 to 0.04). Mean ∆PPTh and SE are presented in Figure 2A for time point and Figure 2B for age group and sex.

For ∆Pain40, the TIME [F(1.687,187.226) = 23.490, *P* < 0.001] and AGE effects were significant [F(1,111) = 11.754, *P* = 0.001]. In addition, the PARADIGM × TIME × AGE × SEX interaction [F(1.661,181.030) = 5.950, *P* = 0.004 was significant. To better interpret the findings, 2 ANOVAs were performed dividing the data by paradigm (CPT, CHP), which was not significant.

#### 3.1.1. Cold-pressor test for ∆Pain40

**Figure 3.**
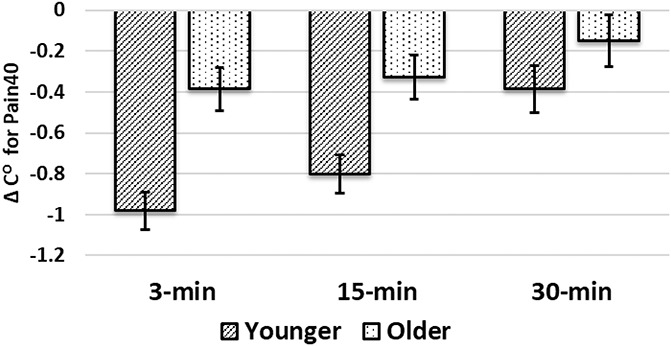
Adjusted mean and SE for ∆Pain40 for the cold-pressor test paradigm for each age group across time. The TIME (*P* = 0.004), AGE (*P* = 0.001), and TIME × AGE (*P* = 0.048) effects were significant. At 3 minutes, the younger group (mean = −0.98) differed from the older group (mean = −0.39). At 15 minutes, the younger group (−0.80) differed from the older group (mean = −0.33). At 30 minutes, the younger group (−0.38) and the older group (−0.15) were not significantly different.

The 3-way ANOVA (TIME × AGE × SEX) for the CPT paradigm resulted in significant effects for TIME [F(1.678,186.308) = 8.436, *P* = 0.004], AGE [F(1,111) = 12.051, *P* = 0.001], and TIME × AGE [F(1.678,184.308) = 3.264, *P* = 0.048]. The AGE × SEX interaction effect was not significant. Figure 3 presents mean and SE each age group across time. Pairwise comparisons at each time point indicate that at 3 minutes, the younger group (mean = −0.98, 95% CI = −1.16 to −0.80) differed from the older group (−0.39, 95% CI = −0.60 to −0.18). At 15 minutes, the younger group (−0.80, 95% CI = −0.99 to −0.62) differed from the older group (−0.33, 95% CI = −0.54 to −0.11). At 30 minutes, the younger group (−0.38, 95% CI = −0.61 to −0.15) did not differ from the older group (−0.15, 95% CI = −0.40 to 0.10).

#### 3.1.2. Contact heat pain for ∆Pain40

**Figure 4.**
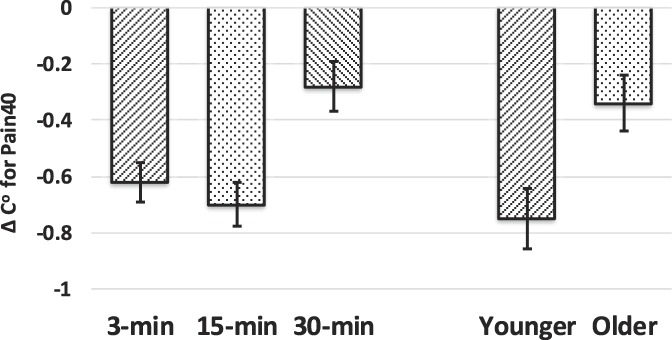
Adjusted mean and SE for ∆Pain40 for the contact heat pain paradigm across time and for each age group. The TIME (*P* = 0.007) and AGE (*P* = 0.001) main effects were significant. Conditioned pain modulation was greater at the 3-minute (mean = −0.62) and 15-minute time points (−0.70) compared to the 30-minute time point (−0.27). Conditioned pain modulation at the 3-minute and 15-minute time points was not significantly different. Collapsed across time, younger adults experienced greater inhibition (mean = −0.80) compared with the older group (−0.34). CPM, conditioned pain modulation.

The 3-way ANOVA (TIME × AGE × SEX) for the CHP paradigm resulted in significant effects for TIME [F(1.879,208.540) = 5.320, *P* = 0.007] and AGE [F(1,111) = 12.108, *P* = 0.001]. The main effect of SEX approached significance [F(1,111) = 2.987, *P* = 0.077]. The AGE × SEX interaction was not significant. Figure 4 presents mean and SE across time and for each age group. Pairwise comparisons at each time point indicated that CPM was greater at the 3-minute (mean = −0.62, 95% CI = −0.76 to −0.48) and 15-minute time points (−0.70, 95% CI = −0.87 to −0.51) compared with the 30-minute time point (−0.27, 95% CI = −0.44 to −0.10). Conditioned pain modulation at 3 minutes and 15 minutes were not significantly different (*P* = 0.09). When collapsed across time, younger adults experienced greater inhibition (−0.80, 95% CI = −0.98 to −0.63) when compared with the older group (−0.34, 95% CI = −0.54 to 0.14).

### 3.2. Conditioned pain modulation and recent pain

Correlations between the CPM indices and the self-reported measures of recent pain are presented in Table 6. For the CPT-∆PPTh, the addition of the pain variables resulted in an increase in *R*^2^ of 14 [∆F (5,108) = 4.179, *P* = 0.002] with the GCPS disability score significant (*P* = 0.014). For the CHP-∆PPTh, the addition of the pain variables resulted in an increase in *R*^2^ of 0.15 [∆F (5,108) = 4.648, *P* = 0.001] with the GCPS number of painful sites significant (*P* = 0.006). For the CPT-∆Pain40 the addition of the pain variables resulted in an increase in *R*^2^ of 0.12 [∆F (5,108) = 3.741, *P* = 0.006] with the GCPS disability score significant (0.006). For the CHP-∆Pain40, the addition of the pain variables resulted in an increase in *R*^2^ of 0.15 [∆F (5,108) = 5.046, *P* < 0.001] with the GCPS disability score significant (*P* = 0.001).

**Table 6:**
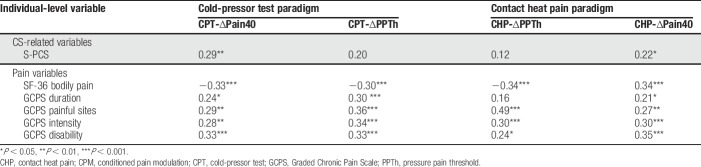
Correlations between individual variables within a domain with zero-order correlations at *P* < 0.05 with the CPM indices.

## 4. Discussion

The study hypothesis for sex differences in both age cohorts was only supported for ∆PPTh as the sex effect was significant in the absence of an age by sex interaction. However, sex differences did not reach significance for either paradigm using ascending suprathreshold heat as the TS. A new finding was that younger males experienced the strongest CPM and older females the weakest, with an age effect larger than the sex effect. Earlier studies have not examined data across age–sex subgroups. The data also revealed duration differences with inhibition of PPTh decaying more quickly than pain induced by ascending suprathreshold heat. In addition, cross-sectional associations were demonstrated between measures of CPM and recent pain.

### 4.1. Methodological considerations

A 2013 consensus meeting called for greater standardization of procedures for performing CPM.^40^ Recommendations included the addition of a second test stimulus and second CPM conditioning protocol to permit comparisons. Avoiding concurrent administration of CS and TS was recommended because sequential test protocols represent a cleaner measure of CPM. Furthermore, the use of contralateral sites on the upper limb and lower limb was suggested. This study followed these recommendations. The rationale for different initial temperatures between males and females is the well-documented sensitivity differences.^7^ Conditioned pain modulation is based on the “pain-inhibition-by-pain” principle and our target is to standardize the level of pain of the CS and not the stimulus intensity. This also eliminated to possibility of only mild CS-related pain in less sensitive males because several studies have shown that CS-repeated pain of >20 is needed for consistent CPM.^9,26^

Reliability of CPM is dependent on the reliability of the TS used. Performing 2 CPM sessions allowed for the calculation of reliability coefficients for the study TS by comparing the baseline values across both sessions. Interclass correlations across sessions for PPTh (*r* = 0.80) and Pain40 (*r* = 0.72) were within the range considered good reliability.^29^ Reaction time was minimized because slow ramping speeds were used for both stimuli. Correlations between baseline levels for PPTh and Pain40 (within-session) were only moderate (shared variance of approximately 16%). However, this is consistent with studies showing pressure threshold and heat pain load on different components after factor analysis.^3,13^

Cold-pressor test has demonstrated better reliability and produces a stronger CPM effect when compared with other modalities.^1,21,27^ This study found that both CPT and CHP resulted in robust inhibition that was similar in magnitude. However, differences in the time course and magnification in circulating substance P, β-endorphin, and several cytokines after CPT compared with CHP have been demonstrated, suggesting differing mechanisms, at least peripherally.^5,33^ Studies have examined whether pain level or stimulus intensity drives CPM with mixed findings.^9,12,21,26^ The current study adjusted the pain intensity to fit each individual's pain sensitivity. No association was found between ∆PPTh or ∆Pain40 and CS testing temperatures or the pain experienced during administration of either CS. It is possible that standardization of the pain experienced attenuated the associations that others have reported.

A systematic review has assessed the reliability of CPM protocols and found considerable variability within and across methodological parameters.^16^ Of the studies reviewed, intrasession reliability ranged from good to excellent and intersession reliability from poor to excellent. Kennedy encouraged future studies to report repeatability for CS and TS when feasible.^16^ Although this study administered 2 standardized TS across both testing sessions, our experimental design examined the effects of CPT and CHP in separate sessions. Nevertheless, the magnitude of the inhibitory effect observed, particularly at 3 minutes (∆PPTh, *r* = 0.61 and ∆Pain40, *r* = 0.53), was similar (eg, repeatable), despite session differences in the CS. At 15 minutes, possible individual differences in the duration of the CPM effect resulted in a less consistent pattern, which dropped further as might be expected by 30 minutes. The magnitude of CPM across paradigms was similar for each time point.

The current findings support the use of short time delays after CS administration. Yarnitsky et al. suggest TS be administered twice, with at least a 10-minute interstimulus interval.^42^ Lewis found a measure of PPTh was reduced at 1, 5, and 10 minutes but not 15 minutes after either CPT or ischemic pain as CS.^21^ The current study found similar duration of inhibition for PPTh, which was shorter compared to inhibition of ascending suprathreshold heat, with none of the age–sex subgroupings experiencing inhibition at 30 minutes with PPTh.

The advantages of ascending suprathreshold pain vs a pain threshold measure have been debated.^17,30^ Threshold involves a decision regarding the change from nonnoxious to noxious, whereas the suprathreshold pain measures require interpretation and may evoke different cognitive and emotional responses.^24,34,35^ Different stimulus modalities may also activate peripheral nerve fibers (A vs C) in a dissimilar manner, thereby stimulating different nociceptive pathways that have varying influences on central nervous system activity.^14^ Furthermore, the longer stronger pain signal associated with Pain40 vs threshold may also elicit differences in central processing. This finding supports CPM as a broad measure of inhibition, suggesting that common and unique mechanisms may be engaged across time and by differing methodologies.^23,24^ This is supported by correlations within CPM paradigms (∆PPTh with ∆Pain40) that were moderate or small for CPT (*r* = 0.36–0.20) and CHP (0.39–0.19) at the same time points. Consequently, it seems that the selection of TS modality/endpoint has a stronger effect on CPM than a choice between CPT and CHP as CS.

### 4.2. Age and sex differences in conditioned pain modulation

As hypothesized, this study supports the existing literature that older adults exhibit diminished descending inhibition. Such age differences have been consistently found across multiple studies that have used similar stimuli and methodology.^6,10,18,31,32,39^ All 6 studies have used cold-water immersion as the CS, and 4 used suprathreshold contact heat.^6,10,31,32^ Lariviere^18^ also used heat threshold, whereas Washington^39^ used electrical and laser thresholds as TS.

Differences in pain between males and females in experimental and clinical settings have been well documented.^7^ However, findings for sex differences in endogenous inhibition during CPM protocols have been mixed^15,28^ with approximately 40% of studies reporting females experiencing less efficient CPM than males. This study found modest differences with females showing diminished descending inhibition compared to males, with similar differences in both age groups. The most consistent finding was that younger males maintained pain inhibition longer than the other 3 sex–age groupings. Testing CPM over 30 minutes allowed for these interesting findings related to sex differences. The current data support a small effect size hypothesis for sex differences with Cohen's D^4^ calculated at 3 minutes resulting in 0.38 and 0.33 for PPTh and 0.10 and 0.29 for Pain40 (CPT and CHP, respectively). Hermans reported that sex differences were more consistent for mechanical pressure threshold measures and our effect sizes fell in that direction.^15^

### 4.3. Associations with recent pain

This study demonstrated cross-sectional associations between measures of CPM inhibition with several measures of recent bodily pain using measures designed for population use, which is seldom reported. Recent pain had a stronger association with the ∆Pain40 variable than with ∆PPTh across both CS. These measures of recent pain represent an aggregate of many acute and prolonged pain experiences, thus suggesting that persons who suffer from pain in general that is not formally classified as “chronic pain” may experience changes in central pain processing.

Omitting persons with GCPS grade 4 level pain and disability reduced the possibility of associations between CPM and greater pain being driven by data at the far end of the distribution because inhibition differences between healthy controls and persons with chronic pain are well established.^22^ The studied sample with 40% reporting no pain and 9% with moderately limiting pain is similar to classification rates reported for enrollees in a large health insurance plan not currently seeking care (50% and 13%, respectively).^36^

### 4.4. Study limitations and strengths

Limitations of this study include the fact that participants were recruited from the community at large, therefore sampling bias is likely, and that measures of bodily pain were remembered pain. It is possible that our findings would have been different had the first post-CS TS been administered closer to the termination of the CS. This study has several strengths, which include a large sample and that participants had a training session with TS administered on a separate day, so that they were comfortable with the stimuli and proficient in rating pain. This likely increased validity and reliability of the participant's ratings, thus reducing error. The associations between baseline pain for both TS were high across sessions and support this assertion. Furthermore, a sequential testing protocol, where the TS were administered after the termination of the CS, eliminated distraction.^40^

## 5. Conclusion

Our hypothesis for sex differences across age cohorts was only supported for ∆PPTh; however, younger males had the strongest CPM across all paradigms and older females had the weakest effect. Overall, the age effect was larger than the sex effect. The data also revealed that younger males maintained pain inhibition longer than the other 3 sex–age groupings. Furthermore, the duration of inhibition for PPTh was shorter when compared with ascending suprathreshold heat. We did not find differences between CPT and CHP as CS; rather both stimuli resulted in robust inhibition. Cross-sectional associations were demonstrated between measures of CPM inhibition with several measures of recent pain and disability, further supporting CPM as an experimental paradigm with clinical utility.

## Disclosures

R.B. Fillingim has received consulting fees, speaking fees, and/or honoraria from WebMD and Algynomics (less than $10,000 each) and owns stock or stock options in Algynomics. The remaining authors have no conflicts of interest to declare.

This research was supported by NIH-NIA Grant R01AG039659.
